# Detection of *Staphylococcus aureus* virulence gene *pvl* based on CRISPR strip

**DOI:** 10.3389/fimmu.2024.1345532

**Published:** 2024-03-08

**Authors:** Li Jin, XiaoFeng Hu, Yuan Tian, MengYa Fang, Xue Dong, YaXuan Jiang, Yao Han, Hao Li, Yansong Sun

**Affiliations:** ^1^ State Key Laboratory of Pathogen and Biosecurity, Beijing Institute of Microbiology and Epidemiology, Beijing, China; ^2^ College of Life Sciences, Fujian Agriculture and Forestry University, Fuzhou, Fujian, China; ^3^ Center for Disease Control and Prevention of Chinese People's Liberation Army, Beijing, China

**Keywords:** *Staphylococcus aureus*, pvl, RAA, CRISPR, ERASE strip

## Abstract

**Introduction:**

*Staphylococcus aureus* (*S. aureus*) is a prominent pathogen responsible for both hospital-acquired and community-acquired infections. Among its arsenal of virulence factors, Panton-Valentine Leucocidin (PVL) is closely associated with severe diseases such as profound skin infections and necrotizing pneumonia. Patients infected with *pvl*-positive *S. aureus* often exhibit more severe symptoms and carry a substantially higher mortality risk. Therefore, it is crucial to promptly and accurately detect *pvl*-positive *S. aureus* before initiating protective measures and providing effective antibacterial treatment.

**Methods:**

In this study, we propose a precise identification and highly sensitive detection method for *pvl*-positive *S. aureus* based on recombinase-assisted amplification and the CRISPR-ERASE strip which we previously developed.

**Results:**

The results revealed that this method achieved a detection limit of 1 copy/μL for *pvl*-positive plasmids within 1 hour. The method successfully identified all 25 *pvl*-positive and 51 *pvl*-negative strains among the tested 76 isolated *S. aureus* samples, demonstrating its concordance with qPCR.

**Discussion:**

These results show that the CRISPR-ERASE detection method for *pvl*-positive *S. aureus* has the advantages of high sensitivity and specificity, this method combines the characteristics of recombinase-assisted amplification at room temperature and the advantages of ERASE test strip visualization, which can greatly reduce the dependence on professional laboratories. It is more suitable for on-site detection than PCR and qPCR, thereby providing important value for rapid on-site detection of *pvl*.

## 1 Introduction

*Staphylococcus aureus* (*S. aureus*) is a Gram-positive bacterium with significant pathogenicity (1), particularly methicillin-resistant *Staphylococcus aureus* (MRSA), which is a leading human pathogen responsible for both hospital- and community-acquired infections worldwide (2). *S. aureus* can colonize various human and animal anatomical sites, including the nasal cavity, gastrointestinal tract (3), skin and other organs (4). When the host’s immune system is compromised, these colonized strains can infect human cells, tissues, and organs, resulting in a myriad of infectious diseases (5), such as local purulent infections, pneumonia (6), pericarditis, pseudomembranous colitis, and even systemic infections such as sepsis. The pathogenicity of *S. aureus* is primarily attributed to a range of toxins (e.g., hemolysin (7), enterotoxin, toxic shock syndrome virus-1) and invasive enzymes (e.g., protease, lipase and hyaluronidase). Notably, Panton-Valentine Leucocidin (PVL) is a pathogenic toxin produced by *S. aureus* (8), which is associated with recurring, often large skin abscesses. *pvl*-positive *S. aureus* skin and soft tissue infections exhibit a higher recurrence rate than *pvl*-negative *S. aureus* infections (9). A German study detected the *pvl* gene in 61.3% (252/411) of *S. aureus* skin and soft tissue infections (10). Patients infected with *pvl*-positive *S. aureus* experience significantly impaired quality of life and mental health (11), with half of the cases being correctly diagnosed at least five months after onset (12). Furthermore, due to a lack of proficiency in diagnosing and treating *pvl*-positive *S. aureus* infections, patients often suffer from increased pain (11). Infections caused by *pvl*-positive *S. aureus* are typically more severe than those caused by *pvl*-negative *S. aureus*: they recur more frequently (with a higher frequency of infection per patient), and result in larger abscesses than those caused by other typical skin pathogens (13, 14). In addition, due to the presence of *pvl* in *S. aureus*, a substantial number of bacteria accumulate in the lungs, triggering a robust inflammatory response. This leads to the formation of lung vacuoles, severe pulmonary edema, and pulmonary hemorrhage. Consequently, *pvl*-positive *S. aureus* is more likely to cause necrotizing pneumonia, with a high mortality rate (15, 16). Michael et al. reported that the mortality of patients with pneumonia caused by *pvl*-positive *S. aureus* within 48 hours of admission was 37%, while the mortality of patients with pneumonia caused by *pvl*-negative *S. aureus* within 48 hours of admission was only 6% (17).

The detection of *S. aureus* virulence factors is primarily conducted via serological tests or PCR techniques, such as double gel immunodiffusion, microslide methods, and latex particle agglutination (18). However, these methods have certain limitations, including the need for specialized laboratory settings, extended incubation times, and intricate procedural requirements (19). Consequently, it is necessary to establish a rapid and reliable method for detecting *S. aureus* virulence factors for the diagnosis of infection and treatment guidance.

Recently, clustered regularly interspaced short palindromic repeats (CRISPR) and CRISPR-associated (Cas) systems have been utilized as significant gene editing tools for virus diagnosis research (20, 21). Zhang Feng’s research group developed the Cas13a-based SHERLOCK platform for the detection of Zika and dengue viruses. The protocol’s outstanding sensitivity plays a crucial role in distinguishing region-specific viral strains (22, 23). Our previous work has led to the development of a CRISPR-ERASE strip that does not entirely rely on specialized detection equipment. It can detect SARS-CoV-2 RNA with a sensitivity of 1 copy/μL and has received approval from the Chinese NMPA(National Medical Products Administration) (24). The CRISPR method has established a complete diagnostic procedure, which can achieve high specificity and sensitivity detection of nucleic acids (23, 25). We developed a sensitive nucleic acid detection technique for the *pvl* virulence genes and *clfA* genes in *S. aureus*.

## 2 Materials and methods

### 2.1 Materials

In this study, all bacterial strains were sourced from laboratory-preserved strains maintained (Chinese People ‘s Liberation Army Center for Disease Control and Prevention, Beijing). These strains encompassed *Staphylococcus aureus*, *Escherichia coli*, *Salmonella and Shigella*. Synthetic plasmids (pUC-SP vector) and DNA fragments, including crRNA, primers, and reporter RNA, were custom-synthesized by Beijing TIANYI-HUIYUAN Biological (Beijing, China). The HiScribe T7 rapid and efficient RNA synthesis kit was purchased from NEW ENGLAND BioLabs (Beijing, China). The RAA detection kit used in the study was procured from Hangzhou ZC Biotechnology Co., Ltd. (Hangzhou, China).

### 2.2 Preparation of nucleic acid

The strains isolated from the hospital and food were cultured in nutrient broth medium and shaken in a 35°C full-temperature oscillator for 18-24 hours. Then DNA was extracted with a bacterial genomic DNA extraction kit to prepare for subsequent experiments. The DNA extraction of the bacterial genomic DNA extraction kit used in this paper was purchased from TIANGEN.

### 2.3 Preparation of crRNA, RAA primers and report RNA

All *clfA* and *pvl* gene sequences of *S. aureus* were obtained from NCBI database (https://www.ncbi.nLm.nih.gov/), and sequence alignment was performed using Mega5.0 software. Then, the conserved sequences matching Cas13a were screened, and the corresponding crRNA was designed as gRNA for subsequent detection. In order to prepare crRNA, single-stranded homologous DNA oligonucleotides with T7 promoter sequences were annealed using primer sequences with complementary T7 primer sequences. Then it was transcribed into crRNA using the HiScribe T7 rapid high-yield RNA synthesis kit. Subsequently, the product crRNA was purified using an RNA purification kit. The optimal crRNA was screened by experiments. Based on the selected optimal crRNA, the corresponding RAA amplification primers and PCR amplification primers were designed using primer5. Fluorescent probe report RNA with sequence FAM-20U-BHQ1. Probe reporter RNA with sequence FAM-20U-Biotin.

### 2.4 RAA amplification

The RAA reaction system was operated in strict accordance with the instructions of the RAA isothermal amplification kit. The RAA system consists of 50 μL: 25 μL buffer, 2.0 μL forward primer (10 μM), 2.0 μL reverse primer (10 μM), 13.5 μL RNase Free Water and 5.0 μL template DNA were added to the detection unit tube containing the detection dry enzyme preparation, and then 2.5 μL LB buffer was added to the detection unit tube. Thoroughly inverted mixing, low speed centrifugal 10 seconds. The test tube was placed in a 39°C incubator for 30 minutes to obtain the amplification product.

### 2.5 Fluorescence CRISPR/Cas13a detection

The isothermal amplification products were mixed with the CRISPR/Cas13a fluorescence detection system. The Cas13a fluorescence detection system consists of 25 μL, including 5 μL RAA amplification products (the above products), 1 μL Cas13a (45 nmol/L), 2 μL NTP Mix (2.5 mmol/L), 1 μL T7 polymerase (2 IU/μL), 0.5 μL HEPES (20 mmol/μL), 0.25 μL MgCl2 (10 mmol/μL), 1 μL RNase Inhibitor (4 IU/μL), 2.5 μL fluorescent reporter RNA (200 nmol/L). 1.5 μL crRNA (280 nmol/L) and 10.75 μL RNase Free Water. The detection system was placed on a fluorescence quantitative PCR instrument at a constant temperature of 37°C for 30 min, once every 2 minutes, and the fluorescence intensity was monitored in real time.

### 2.6 CRISPR-ERASE nucleic acid test strip detection

The isothermal amplification products were mixed with the CRISPR-ERASE strip detection system. The CRISPR-ERASE strip detection system consisted of 50 μL, including 5 μL RAA amplification product (the above product), 2 μL Cas13a (80 ng/L), 4 μL NTP Mix (2.5 mmol/L), 2 μL RNase Inhibitor (1.6 IU/μL), 1 μL HEPES (20 mmol/μL), 0.5 μL MgCl_2_ (10 mmol/μL), 1 μL RNase Inhibitor (2 IU/μL). 5μL FAM-20U-Biotin (200 nmol/L), 3μL crRNA (280 nmol/L) and 26.5 μL RNase Free Water. After incubation at 37°C for 30 minutes in a metal bath, the entire reaction system was transferred to the ERASE strip with a pipette, and the test results were read by the naked eye after waiting for 3-5 minutes.

### 2.7 Sensitivity experiment

The synthetic plasmid was obtained by cloning the *clfA* and *pvl* fragments into the pUC-SP vector. The pUC-SP vector was modified from pUC57 (26). Synonymous mutations were used to eliminate the common sticky end sites in the polyclonal enzyme digestion sites, and only the common flat end sites were retained, which could meet the needs of the only enzyme digestion sites at both ends of the gene. After plasmid extraction and purification, NanoPhotometer N60 spectrophotometer (IMPLEN, DEU) was used to determine the concentration. The solution was diluted to a concentration of 10^9^-10^-1^ copies/μL, from which 10-fold gradient dilution was used as a detection template for LoD.

### 2.8 Sensitivity evaluation of fluorescence quantitative PCR detection

The *clfA* and *pvl* genes were detected by fluorescence quantitative PCR kit (TaKaRa) after a 10-fold gradient dilution. The qPCR system was: 12.5 μL TB Green Premix ExII (2 ×), 1μL PCR reverse primer (final concentration of 0.4 μmol/L), 1μL PCR forward primer (final concentration of 0.4 μmol/L), 8.5 μL RNase Free Water, 2 μL target DNA, a total of 20 μL system. It was placed on a fluorescence quantitative PCR instrument, pre-denaturation at 95°C for 30 seconds, and then denaturation at 95°C for 5 seconds; at 60°C, 40 cycles of 1 min were repeated, and the fluorescence signal was collected at the end of each cycle.

### 2.9 Specificity determination experiment

We specifically selected commonly encountered bacteria in clinical infections as subjects for our experiments. Nucleic acids were extracted from *Staphylococcus aureus*, *Escherichia coli, Salmonella* and *Shigella*, with the extracted DNA serving as the template for subsequent processes, including RAA amplification, CRISPR fluorescence detection, and CRISPR-ERASE strip detection.

### 2.10 Isolated sample detection

The Chinese People ‘s Liberation Army Center for Disease Control and Prevention provided a total of 76 isolated samples. The samples were first subjected to bacterial culture, and then detected by four different methods: PCR, fluorescence quantitative PCR, RAA-Cas13a fluorescence and CRISPR-ERASE. PCR assays were performed in 50 μL reaction mixture, including 25 μL EXTaq (purchased from TaKaRa), 1 μL forward primer (10 μM), 1 μL reverse primer (10 μM), 5 μL extracted template DNA and 18 μL ddH_2_O. PCR assays were performed using the following thermal cycle process: pre-denaturation at 95°C for 5 minutes, followed by 28 cycles with the following parameters: denaturation at 95°C for 30 seconds, annealing at 60°C for 30 seconds, extension at 72°C for 15 seconds, and finally extension at 72°C for 10 minutes. The above experiments are repeated twice to avoid experimental errors and ensure the experimental results.

### 2.11 Statistical analysis

The data analysis was conducted using GraphPad Prism 8.0.1. Fluorescence values were presented as the mean ± standard deviation (SD), with the mean fluorescence value derived from three independent experiments. Multiple group comparisons were performed utilizing one-way analysis of variance (ANOVA), and the difference was significant when *p < 0.05.

## 3 Results

### 3.1 Design and screening of CRISPR-ERASE system

**Figure 1:**
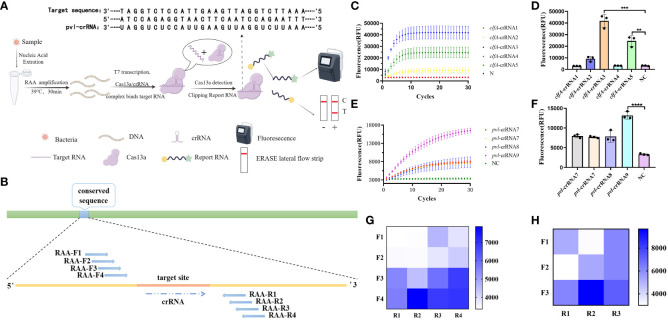
Design and screening of CRISPR-ERASE system. Negative control (NC) refers to replacing the template with H_2_O. **(A)** Principle of CRISPR-ERASE detection system. The detection process of CRISPR-ERASE detection system. **(B)** The schematic diagram of the design includes paired RAA primers and crRNA. **(C)** Screening the best crRNA of *clfA* gene. The dynamic fluorescence curves of different crRNAs in CRISPR fluorescence detection of the same concentration of *clfA* positive plasmids without amplification. **(D)** The fluorescence values of different crRNAs designed for the *clfA* gene were compared after 30 minutes of CRISPR fluorescence detection reaction, **p < 0.01; ***p < 0.001; ****p < 0.0001. The data were expressed as mean ± SD (n = 3). **(E)** Screening the best crRNA of *pvl* gene. The dynamic fluorescence curves of different crRNAs in CRISPR fluorescence detection of the same concentration of *pvl* positive plasmids without amplification. **(F)** Comparison of screening the fluorescence value of *pvl* gene after 30 minutes of CRISPR fluorescence detection reaction. **(G)** The fluorescence values produced after 60 minutes of CRSIPR fluorescence detection reaction with *pvl*-crRNA9 and different RAA amplification primer pairs of *pvl* gene. The data were expressed as mean ± SD (n = 3). **(H)** The fluorescence values produced with *clfA*-crRNA3 and different primer combinations of *clfA* gene after 60 minutes of CRISPR fluorescence detection reaction.

The process and fundamental principles of CRISPR-ERASE-based detection are schematically depicted in **Figure 1A**. This detection process can be divided into three key steps. In the first step, the initial sample undergoes a pretreatment procedure, during which DNA is extracted to serve as the substrate for subsequent steps. This is followed by Step II, where nucleic acids are amplified through recombinase-assisted amplification (RAA). Finally, in Step III, a signal is generated based on Cas13a. As illustrated in **Figure 1B**, nine crRNAs were designed within the selected conserved sequences, namely *clfA*-crRNA1, *clfA*-crRNA2, *clfA*-crRNA3, *clfA*-crRNA4, *clfA*-crRNA5, *pvl*-crRNA6, *pvl*-crRNA7, *pvl*-crRNA8, and *pvl*-crRNA9. Subsequently, upstream and downstream primers of RAA were designed on both sides of each crRNA. To employ the CRISPR-ERASE system for detecting clinical *pvl*-positive *Staphylococcus aureus* strains, sequence alignment of *clfA* and *pvl* sequences was performed to identify conserved target areas and design crRNAs. This was followed by the detection of positive plasmids of the same concentration. The screening results (**Figures 1C–F**) indicate that the fluorescence value of *clfA*-crRNA3 reached 41817.4433 ± 5668.25 a.u. at 30 min, which was 38552.4266 ± 5,401.5313 a.u. higher than that of the negative control group (3262.0167 ± 266.7187 a.u.). The fluorescence value of *pvl*-crRNA9 reached 13160.5567 ± 928.5862 a.u. at 30 min, while the negative control group registered 3345.2867 ± 218.198 a.u. Consequently, *clfA*-crRNA3 and *pvl*-crRNA9 were selected for subsequent experiments.

To identify efficient isothermal amplification primers for achieving high-sensitivity detection, we combined primer pairs targeting the conserved sequence of the gene in pairs, and amplified plasmids with a concentration of 10^0^ copy/μL. The results (**Figures 1G, H**) demonstrate that the fluorescence value of *pvl*-F4R2 can reach 7863.05 ± 117.3901 a.u. at 60 minutes of reaction, while the negative control group is 3365.3433 ± 55.9647 a.u. The fluorescence value of *clfA*-F2R3 reach 9673.2933 ± 742.6073 a.u. at 60 minutes, which is 6,616.04 ± 624.6737 a.u. higher than that of the negative control group (3057.2533 ± 117.9336 a.u.). These two pairs of primers are used for subsequent experiments.

### 3.2 Sensitivity evaluation based on CRISPR-ERASE detection

**Figure 2:**
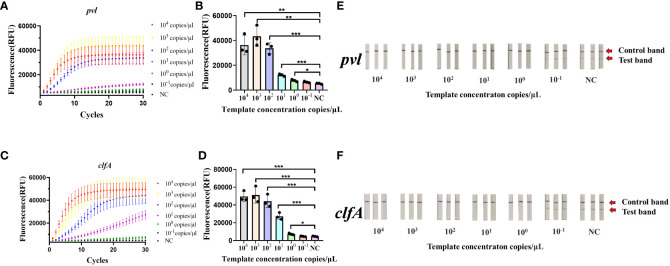
Sensitivity of the CRISPR-ERASE Detection System for *pvl* and *clfA* DNA. Negative control (NC) utilized RNase-free water. **(A)** The fluorescence curve of gradient diluted plasmid containing *pvl* gene was detected by CRSIPR fluorescence after 60 min. **(B)** Comparison of fluorescence values produced by CRSIPR fluorescence detection of *pvl* plasmid after 60 minutes, *p < 0.05; **p < 0.01; ***p < 0.001. The data were expressed as mean ± SD (n = 3). **(C)** The fluorescence curve of the gradient diluted *clfA* plasmid was detected by CRISPR fluorescence after 60 min. **(D)** Comparing the fluorescence value of *clfA* plasmid detected by CRISPR fluorescence method after reaction for 60 min. **(E)** The results of CRISPR-ERASE strips at different *pvl* plasmid concentrations (10^4^ ~10^-1^ copies/μL). Test band disappeared and control band was visible meaning that the test result is positive. Repeat each result three times. **(F)** The results of gradient dilution of *clfA* plasmid were detected by CRISPR-ERASE for 60 minutes.

The synthetic plasmids harboring the *clfA* and *pvl* genes were subjected to a fixed concentration gradient of 10-fold dilution (10^4^ copies/μL to 10^-1^ copies/μL), followed by the use of these gradient-diluted *clfA* and *pvl* plasmids as templates. A negative control group utilizing enzyme-free water was established for the RAA isothermal amplification, followed by subsequent fluorescence detection and test strips. As illustrated by the results of fluorescence detection (**Figures 2A–D**), a significant increase in fluorescence values was observed in the four experimental groups (10^4^ copies/μL to 10^0^ copy/μL) 60 minutes post-reaction initiation, with these values being statistically distinct from those of the negative control group (*p < 0.05). When a concentration of 10^0^ copy/μL of *pvl* plasmid was detected, the average fluorescence value reached 7750.6 ± 898.727 a.u., exhibiting statistical difference from the negative control group; correspondingly, when 10^0^ copy/μL *clfA* plasmid was detected, the average fluorescence value reached 7249.0833 ± 1090.3527 a.u. The detection limit of the *clfA* gene and virulence gene *pvl* fluorescence detection method based on CRISPR was determined to be 10^0^ copy/μL. The sensitivity of 10^0^ copy/μL can also be achieved by adding ERASE test strip (**Figures 2E, F**). The copy number of plasmid was 1 copy/μL in 10 times of detection, and the results of ERASE test strip were positive. It is proved that ERASE test paper has good stability (**Supplementary Figure S1**). Subsequently, the synthetic plasmids containing *clfA* and *pvl* genes underwent PCR amplification and fluorescence quantitative PCR detection, respectively. As revealed by agarose gel electrophoresis (**Supplementary Figures S2**, **S3**), detection was not possible when the concentration was less than 10^5^ copies/μL, while the detection limit of qPCR was established at 10^0^ copy/μL (**Supplementary Figure S4**).

### 3.3 The specificity of CRISPR-ERASE detection system

**Figure 3:**
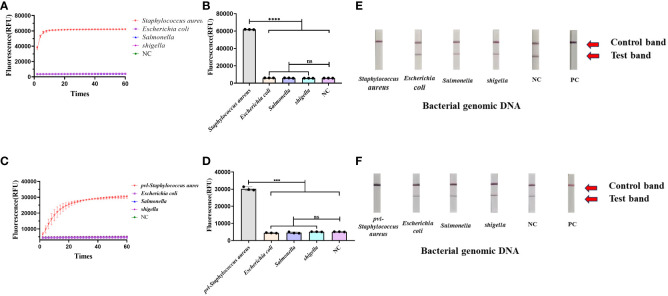
Determine the specificity of the CRISPR-ERASE detection system. The data were expressed as mean ± SD (n = 3). The negative control (NC) was treated with non-enzymatic water. ***p < 0.001; ****p < 0.0001; ns, no significance. **(A)** CRISPR-ERASE system was used to detect the fluorescence curves of *clfA g*ene in different clinical strains after 60 min. **(B)** CRISPR-ERASE was used to detect the final fluorescence value of *clfA* gene in different clinical bacterial samples after 30 min of reaction. **(C)** The fluorescence curves of *pvl* gene in different clinical strains were detected by CRISPR-ERASE system after 60 min. **(D)** CRISPR-ERASE was used to detect the final fluorescence value of *pvl* gene in different clinical bacterial samples after 30 min of reaction. **(E)** CRISPR-ERASE was used to detect the *clfA* gene of different strains, and the results of the test strip were displayed after 30 minutes. **(F)** CRISPR-ERASE was used to detect the *pvl* gene of different strains, and the results of the test strip were displayed after 30 minutes.

A variety of bacterial strains were assessed using the CRISPR-ERASE method. The findings, observed after a 30-minute detection period, revealed a significant difference in fluorescence values between *Staphylococcus aureus* and the negative control group (****p < 0.0001), as indicated in **Figures 3A, B**. *Escherichia coli*, *salmonella*, *shigella*, and the negative control group test results were negative. Additionally, the results from the ERASE strips demonstrated that the detection line corresponding to *Staphylococcus aureus* was eliminated, indicating a positive outcome. In contrast, the test results for other strains remained negative (**Figure 3E**).

The CRISPR-ERASE method was utilized for the detection of various bacterial strains. Following a 30-minute detection period, the final fluorescence values exhibited statistical significance exclusively in the case of *Staphylococcus aureus* containing *pvl* when compared to the negative control (***p = 0.0007< 0.001), as shown in (**Figures 3C, D**). The test results for *Escherichia coli*, *Salmonella*, *Shigella*, and the negative control group remained negative. Concurrently, the results from the ERASE strips revealed that only the detection line corresponding to *Staphylococcus aureus* containing *pvl* exhibited elimination, signifying a positive result. Meanwhile, the test outcomes for other bacterial strains yielded negative results (**Figure 3F**).

### 3.4 Validation of real sample evaluation

**Figure 4:**
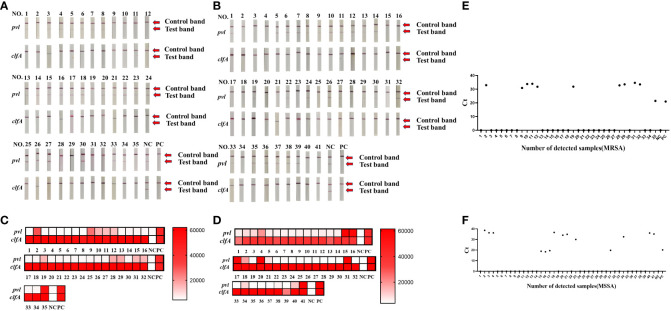
Clinical validation of CRISPR-ERASE detection system to detect *pvl* gene and *clfA* gene in isolated *S. aureus* samples. Sample numbers 1-35 represent 35 MRSA isolates. Sample numbers 1-41 represent 41 strains of isolated MSSA samples. **(A)** CRISPR-ERASE system detection results for 35 MRSA isolated samples, including the detection results of *pvl* gene and *clfA* gene. **(B)** The results of CRISPR-ERASE detection of *clfA* gene and *pvl* gene in 41 MSSA isolated samples. **(C)** The fluorescence value of CRISPR fluorescence detection for 35 MRSA isolated samples, and results are indicated above the heat map. Red indicates that the gene detected in the sample is positive, and the shadow of the color is positively correlated with the fluorescence value. White indicates that the gene tested in the sample is negative, and the depth of red is negatively correlated with the fluorescence value. Positive control (PC) and negative control (NC). Both *clfA* and *pvl* tests were positive, indicating that the sample was *Staphylococcus aureus* containing the virulence gene *pvl*. **(D)** The fluorescence value of CRISPR fluorescence detection for 41 MSSA isolated samples, and results are indicated above the heat map. **(E)** The qPCR assay was employed to determine the Cycle Threshold (Ct) values of the *pvl* gene in isolated MRSA samples. A Ct value of 0 was considered as indicative of a negative result, while other Ct values were interpreted as positive. **(F)** Ct value of the *pvl* gene in isolated MSSA samples was determined through qPCR analysis.

To assess the efficacy of the CRISPR-ERASE detection system in isolated samples, we employed qPCR, PCR, and the CRISPR-ERASE detection system to analyze 35 MRSA strains and 41 MSSA strains, totaling 76 isolated *Staphylococcus aureus* specimens. Agarose gel electrophoresis results revealed that three of these *Staphylococcus aureus* strains contained the *pvl* gene. This confirmed the presence of the *pvl* gene in these three strains, as the sensitivity of the agarose gel electrophoresis experiment was 10^5^ copies/μL. Due to its lower sensitivity, PCR could not detect the *pvl* gene at such low levels, further affirming that these three strains were indeed strongly positive for the *pvl* gene (see **Supplementary Figures S5**, **S6**). Simultaneously, all 76 strains underwent analysis using the CRISPR-ERASE detection system. The results indicated that all 76 strains were *Staphylococcus aureus*, with 25 of them harboring the *pvl* gene. Among the 35 MRSA strains, 11 contained the *pvl* gene, while 14 of the 41 MSSA strains exhibited the presence of the *pvl* gene (as depicted in **Figures 4A–D**). This finding underscores the consistency between *pvl*-positive *S. aureus* detection using the CRISPR-ERASE system and the results obtained from qPCR and PCR. To further validate our method, we calculated the Positive Predictive Agreement (PPA) and Negative Predictive Agreement (NPA), as summarized in **Table 1**. These metrics indicate that when compared to qPCR results (**Figures 4E, F**), our method achieved a match: 25/25 for *pvl*-positive and 51/51 for *pvl*-negative samples.

**Table 1:** The table shows the consistency between the CRISPR-ERASE and qPCR assay.

Method	CRISPR-ERASE									
qPCR^1^	MRSA^2^	*pvl*-positive	*pvl*-negative	Total	MSSA^3^	*pvl*-positive	*pvl*-negative	Total	PPA^4^(95%CI)	NPA^5^(95%CI^6^)
	*pvl*-positive	11	0	11	*pvl*-positive	14	0	14	100%	100%
	*pvl*-negative	0	24	24	*pvl*-negative	0	27	27	100%	100%
	Total	11	24	35	Total	14	27	41	100%	100%

qPCR^1^, fluorescence quantitative polymerase chain reaction; MRSA^2^, methicillin-resistant *Staphylococcus aureus*; MSSA^3^, methicillin-sensitive *Staphylococcus aureus*; PPA^4^, positive predictive agreement; NPA^5^, negative predictive agreement; CI^6^, confidence interval.

## 4 Discussion

In this study, we developed a method for detecting *Staphylococcus aureus* with a *pvl* virulence gene, successfully combines CRISPR-Cas13a with Recombinase-Aided Amplification (RAA) and visualized the results using the ERASE strip. This approach enabled precise identification and highly sensitive detection of Staphylococcus aureus strains carrying the *pvl* virulence gene in isolated samples. The results obtained with the CRISPR-ERASE strip demonstrated strong concurrence with the qPCR and PCR results for 76 isolated samples. Importantly, the CRISPR-ERASE strip method offers a significant advantage by eliminating the need for specialized equipment or highly trained personnel in the process of detecting *Staphylococcus aureus* with virulence gene *pvl*. This capability opens up new possibilities for on-site testing.

Studies by Michael et al. (17) have shown that the prognosis of patients with *pvl*-positive and *pvl*-negative is very different, and the diagnosis delay and high recurrence rate are the main reasons for the recurrence of the patient’s condition. In view of this, a rapid and sensitive method to identify *pvl* in *Staphylococcus aureus* strains is of great importance. In this study, we established and evaluated a novel method for detecting *Staphylococcus aureus* and its virulence gene *pvl* based on CRISPR-ERASE. The gel electrophoresis results following PCR amplification in this study revealed a detection limit of 10^5^ copies/μL. In contrast, the CRISPR-ERASE detection system achieved a lower Limit of Detection (LoD) of 1 copy/μL within 60 minutes. Therefore, the new method we established can effectively detect *Staphylococcus aureus* and its virulence gene *pvl*.

We utilized CRISPR-ERASE technology to detect *Staphylococcus aureus* and its virulence gene *pvl*, achieving high sensitivity, high specificity, and visualization. In recent years, nucleic acid isothermal amplification technology has been applied to the detection of pathogens of infectious diseases due to its specificity, simplicity and efficiency, and plays an increasingly important role in promoting the on-site detection and control of pathogens (27). Compared to other amplification techniques, RAA/RPA amplification requires only ambient temperature (37-42°C) and a single cup of hot water to achieve amplification, eliminating the need for complex thermal cycling processes and professional laboratory personnel. This facilitates further on-site testing possibilities (28). The difference between RAA and RPA is that RAA is a recombinase obtained from bacteria or fungi to replace the phage recombinase that is difficult to obtain in RPA technology. The advent of nucleic acid detection technology based on CRISPR-Cas has opened new avenues for pathogen detection (29–31). In contrast to traditional methods, the CRISPR-Cas system imposes minimal demands on sample quality and exhibits robust resistance to interference. Additionally, it can be seamlessly integrated with rapid sample preprocessing techniques, enabling on-site nucleic acid extraction without specialized equipment (32). Notably, recent advancements have led to the development of a CRISPR-Cas13a-based nucleic acid visualization rapid detection technology tailored for lateral-flow test strip applications. This innovation involves the modification of biotin and the small molecule antigen FAM at both ends of the reporter RNA (33, 34). The ERASE strip, originating from these developments, has been successfully employed for the rapid detection of SARS-CoV-2. Unlike traditional methods, the ERASE strip approach (24) offers portability and rapidity, allowing for real-time visual detection with the naked eye at the point of testing. This detection system enables convenient visual identification of specific nucleic acids, including pathogens and gene mutations, among other applications. Our established detection method combines RAA-CRISPR and ERASE techniques. Compared to the PCR method, our approach possesses the advantages of RAA’s simplicity and ERASE’s visualization capabilities, further realizing the potential for on-site detection. We compared the CRISPR-ERASE detection method with the existing Real-time PCR assay (35), Pentaplex PCR (36) and Whole-Genome Sequencing (WGS) (37) detection of virulence gene *pvl* (**Table 2**). The CRISPR-ERASE detection method we established has good sensitivity and specificity.

**Table 2:** Comparison of CRISPR-ERASE detection method and existing detection methods.

Detect method	Experimental period	Sensitivity	Specificity
CRISPR-ERASE	1 h	1 copy/μL	100%
Real-time PCR assay	1 h	18 copies/reaction (7.2 copies/μL)	100%
Pentaplex PCR	4 h	10 ng DNA (10^4^ CFU^1^/mL)	100%
Whole-Genome Sequencing (WGS)	1 week	100%	100%

CFU^1^, colony forming units.

Although our established CRISPR-ERASE method demonstrates high sensitivity and specificity in the detection of *Staphylococcus aureus* and its virulence gene *pvl*, it is not without limitations. One such constraint is the prerequisite for nucleic acid extraction from bacteria, a process that necessitates specialized instruments, thereby hindering the feasibility of on-site detection. Nonetheless, it is important to note that nucleic acid lysates are now available, and with ongoing technological advancements, the goal of on-site detection might be achievable in the future.

## Data availability statement

The original contributions presented in the study are included in the article/**Supplementary Materials**, further inquiries can be directed to the corresponding author/s.

## Author contributions

LJ: Conceptualization, Data curation, Methodology, Validation, Visualization, Writing – original draft. XH: Resources, Writing – review and editing. YT: Visualization, Writing – review and editing. MF: Data curation, Writing – review and editing. XD: Data curation, Writing – review and editing. YJ: Data curation, Writing – review and editing. YH: Funding acquisition, Writing – review and editing. HL: Conceptualization, Resources, Supervision, Writing – review and editing. YS: Conceptualization, Funding acquisition, Resources, Writing – review and editing.
