# Supplementary material for: Detection of Staphylococcus aureus virulence gene pvl based on CRISPR strip
Source: Front Immunol. 2024 Mar 8;15:1345532. doi: 10.3389/fimmu.2024.1345532 (PMC10957627; doi:10.3389/fimmu.2024.1345532)
Supplement: Supplementary file 1 [file DataSheet_1.docx]

Supplementary Material

**Table S1.** crRNA sequences (The position of the mark red represents the target sequence)

| crRNA name | Sequences (5′–3′) |
| --- | --- |
| *clfA*-crRNA1 | GGGAUUUAGACUACCCCAAAAACGAAGGGGACUAAAACGUUGAAACAUUUUCCGCAUUUGUAGAAU |
| *clfA*-crRNA2 | GGGAUUUAGACUACCCCAAAAACGAAGGGGACUAAAACUUGUUGAAACAUUUUCCGCAUUUGUAGA |
| *clfA*-crRNA3 | GGGAUUUAGACUACCCCAAAAACGAAGGGGACUAAAACGCGUUGUUGAAACAUUUUCCGCAUUUGU |
| *clfA*-crRNA4 | GGGAUUUAGACUACCCCAAAAACGAAGGGGACUAAAACAACAUUUUCCGCAUUUGUAGAAUUUUGA |
| *clfA*-crRNA5 | GGGAUUUAGACUACCCCAAAAACGAAGGGGACUAAAACUGAAACAUUUUCCGCAUUUGUAGAAUUU |
| *pvl*-crRNA6 | GGGAUUUAGACUACCCCAAAAACGAAGGGGACUAAAACAAUAAAUUCUGGAUUGAAGUUACCUCUG |
| *pvl*-crRNA7 | GGGAUUUAGACUACCCCAAAAACGAAGGGGACUAAAACAUAAAUUCUGGAUUGAAGUUACCUCUGG |
| *pvl*-crRNA8 | GGGAUUUAGACUACCCCAAAAACGAAGGGGACUAAAACUAAAUUCUGGAUUGAAGUUACCUCUGGA |
| *pvl*-crRNA9 | GGGAUUUAGACUACCCCAAAAACGAAGGGGACUAAAACAAAUUCUGGAUUGAAGUUACCUCUGGAU |
| T7 primer | TAATACGACTCACTATAG |
| Template *clfA*-crRNA1 | ATTCTACAAATGCGGAAAATGTTTCAACGTTTTAGTCCCCTTCGTTTTTGGGGTAGTCTAAATCCC |
| Template *clfA*-crRNA2 | TCTACAAATGCGGAAAATGTTTCAACAAGTTTTAGTCCCCTTCGTTTTTGGGGTAGTCTAAATCCC |
| Template *clfA*-crRNA3 | ACAAATGCGGAAAATGTTTCAACAACGCGTTTTAGTCCCCTTCGTTTTTGGGGTAGTCTAAATCCC |
| Template *clfA*-crRNA4 | TCAAAATTCTACAAATGCGGAAAATGTTGTTTTAGTCCCCTTCGTTTTTGGGGTAGTCTAAATCCC |
| Template *clfA*-crRNA5 | AAATTCTACAAATGCGGAAAATGTTTCAGTTTTAGTCCCCTTCGTTTTTGGGGTAGTCTAAATCCC |
| Template *pvl*-crRNA6 | CAGAGGTAACTTCAATCCAGAATTTATTGTTTTAGTCCCCTTCGTTTTTGGGGTAGTCTAAATCCC |
| Template *pvl*-crRNA7 | CCAGAGGTAACTTCAATCCAGAATTTATGTTTTAGTCCCCTTCGTTTTTGGGGTAGTCTAAATCCC |
| Template *pvl*-crRNA8 | TCCAGAGGTAACTTCAATCCAGAATTTAGTTTTAGTCCCCTTCGTTTTTGGGGTAGTCTAAATCCC |
| Template *pvl*-crRNA9 | ATCCAGAGGTAACTTCAATCCAGAATTTGTTTTAGTCCCCTTCGTTTTTGGGGTAGTCTAAATCCC |

**Table S2.** Primers sequences (The position of the mark red represents the target sequence)

| Primer name | Sequences (5′–3′) | Amplicon length (bp) |
| --- | --- | --- |
| RAA-*clfA*-F-2 | AATTCTAATACGACTCACTATAGGGATCAAACAAGTAATGAAACGACTTCTAATG | 170 |
| RAA-*clfA*-R-3 | GGAGCTGATTCATTGTTTGAAGGTGTTGCT |  |
| RAA-*pvl*-F-4 | AATTCTAATACGACTCACTATAGGGGGCTCAAGACAAAGCAACTTAAATGCTGGA | 149 |
| RAA-*pvl*-R-2 | GCAGCGTTTTGTTTTCGAGATAGGACACCA |  |
| PCR-*clfA*-F | AATCAAACAAGTAATGAAACGAC | 144 |
| PCR-*clfA*-R | CATTGTTTGAAGGTGTTGCT |  |
| PCR- *pvl*- fw | AAATGCTGGACAAAACTTCTTGG | 108 |
| PCR- *pvl*- rev | TTTGCAGCGTTTTGTTTTCG |  |

**Table S3.** Reference plasmid sequences

| Plasmid name | Sequences (5′–3′) | Length (bp) |
| --- | --- | --- |
| Reference plasmids containing *clfA* | ACCGGCAACAACTCAATCAAGCAATACAAATGCGGAGGAATTAGTGAATCAAACAAGTAATGAAACGACTTCTAATGATACTAATACAGTATCATCTGTAAATTCACCTCAAAATTCTACAAATGCGGAAAATGTTTCAACAACGCAAGATACTTCAACTGAAGCAACACCTTCAAACAATGAATCAGCTCCACAGAGTACAGATGCAAGTAATAAAGATGTAGTTAATCAAGCGGTTAATACAAGTGCGCCTAGAAAGAGAGCATTTAGTTTAGCGGCTGTAGCTGCAGATGCACCGGC | 300 |
| Reference plasmids containing *pvl* | TCAGGTGGAGGTAATGGTTCAAAATCTTTTTCAGAGACAATTAACTATAAACAAGAAAGCTATAGAACTAGCTTAGATAAAAGAACTAATTTCAAAAAAATTGGTTGGGATGTTGAAGCACATAAAATTATGAATAATGGTTGGGGACCATATGGCAGAGATAGTTATCATTCAACTTATGGTAATGAAATGTTTTTAGGCTCAAGACAAAGCAACTTAAATGCTGGACAAAACTTCTTGGAATATCACAAAATGCCAGTGTTATCCAGAGGTAACTTCAATCCAGAATTTATTGGTGTCCTATCTCGAAAACAAAACGCTGCAAAAAAATC | 332 |


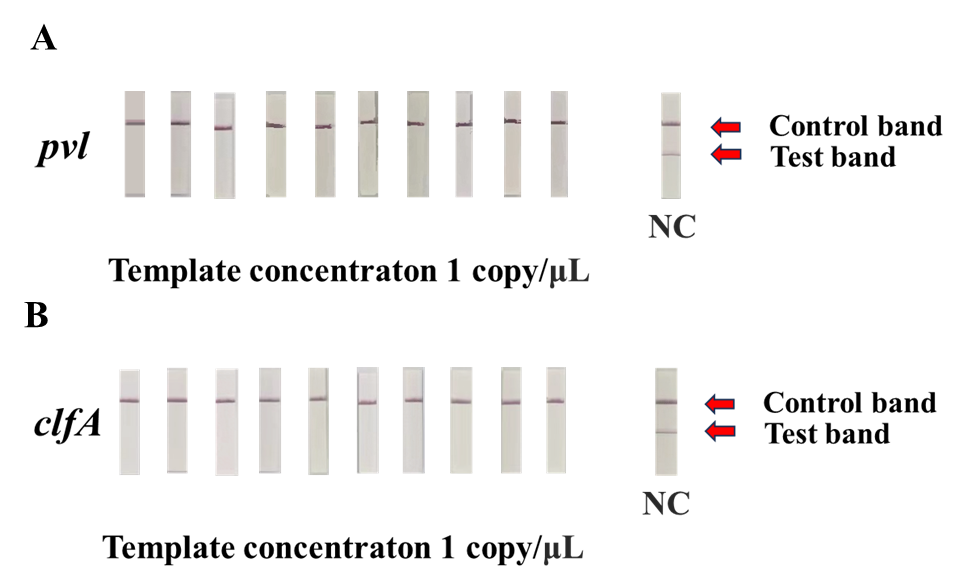


**Figure S1.** The Stability results of plasmid containing *pvl* gene or *clfA* gene by CRISPR-ERASE.

**
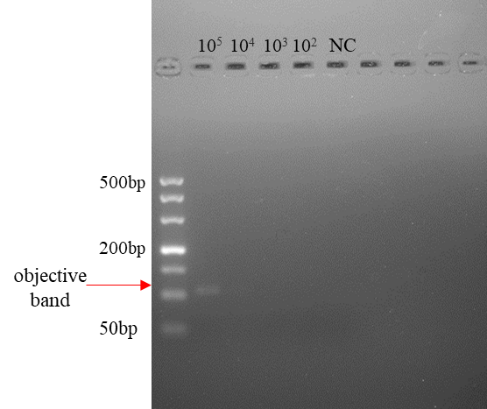
**

**Figure S2.** The results of agarose gel electrophoresis of *clfA* target gene amplified by PCR at each dilution.

**
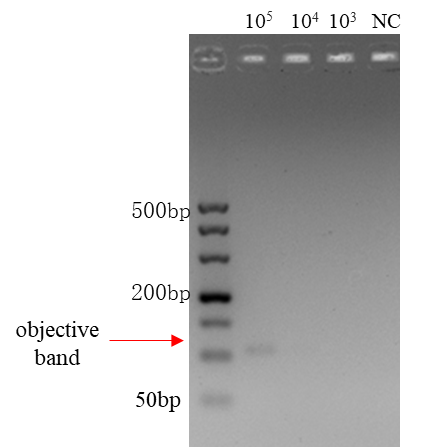
**

**Figure S3.** The results of agarose gel electrophoresis of *pvl* target gene amplified by PCR at each dilution.

**
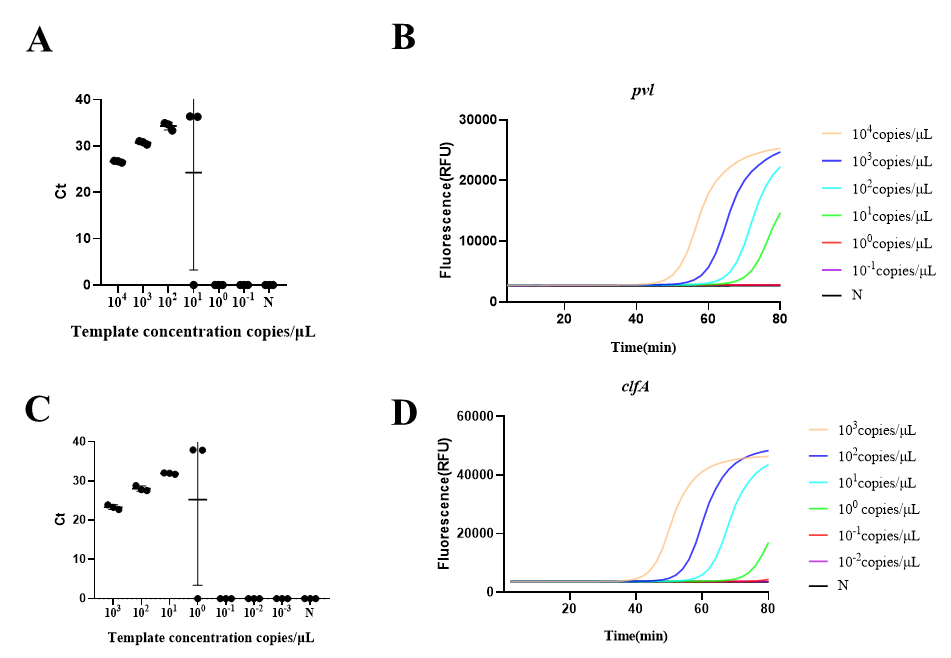
**

**Figure S4.** Sensitivity of the qPCR Detection System for *pvl* and *clfA* DNA. (A) The plasmid containing *pvl* gene was detected by fluorescence quantitative PCR, and the Ct value of the system was detected. (B) Fluorescence quantitative PCR detection system fluorescence report curve of detected *pvl* gene. (C) The plasmid containing *clfA* gene was detected by qPCR, and the Ct value of the detection system was detected. (D) qPCR detection system fluorescence report curve of detected *clfA* gene.

**
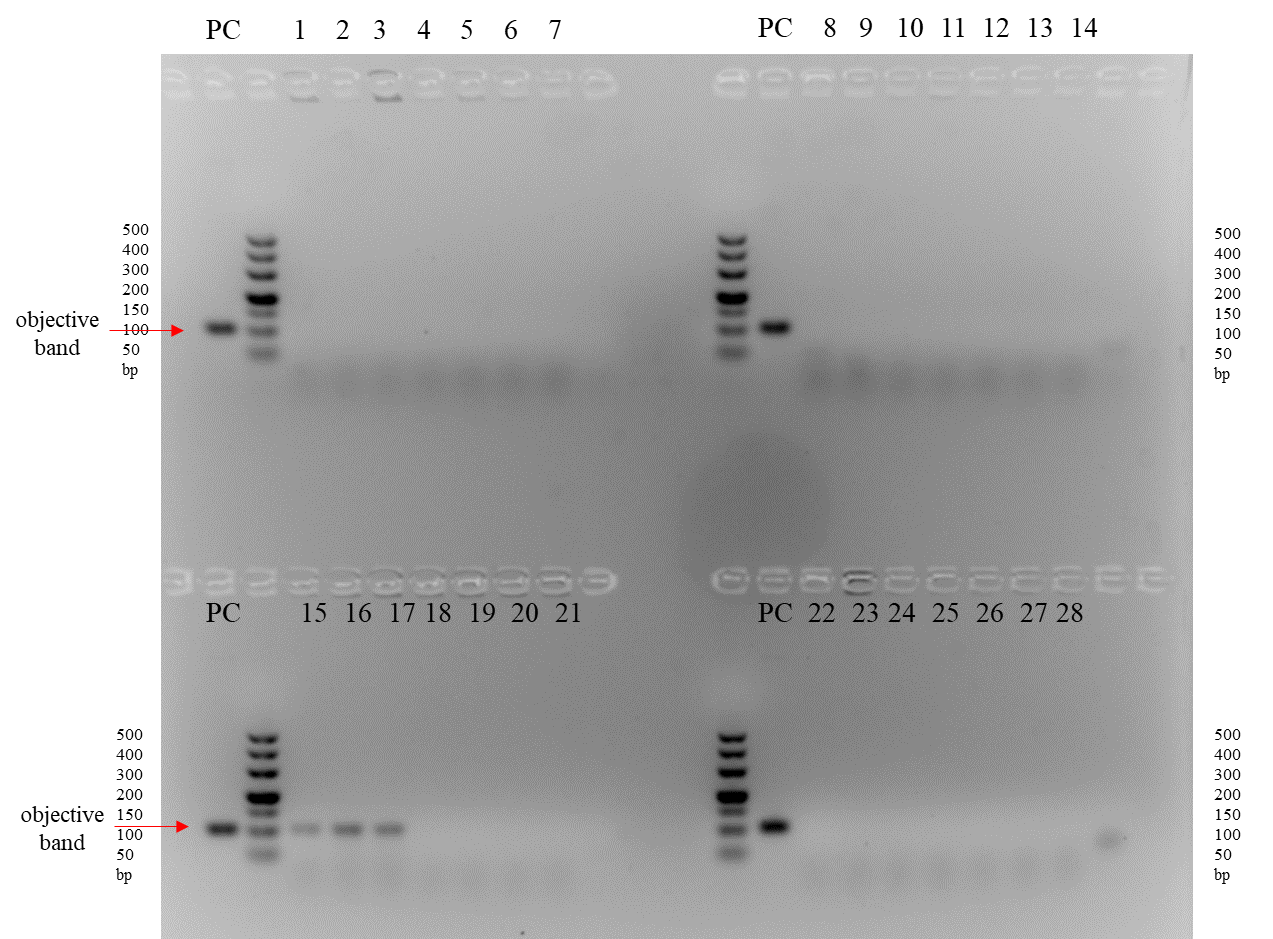
**

**
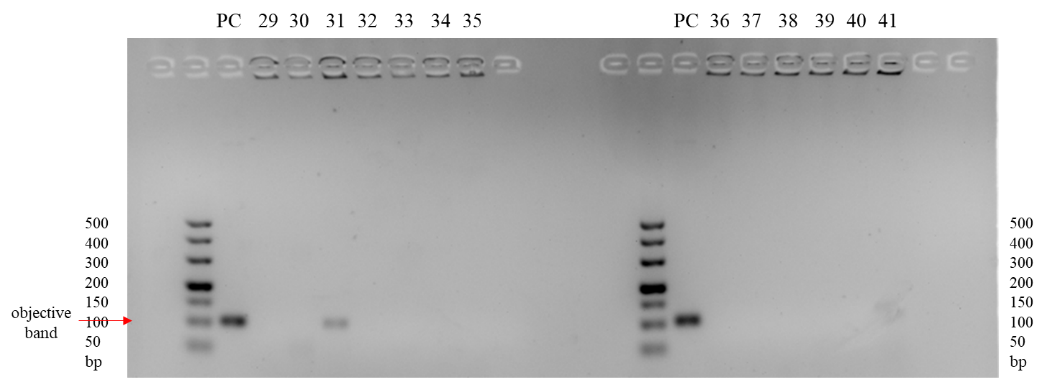
**

**Figure S5.** The results of agarose gel electrophoresis of MSSA isolated samples *pvl* target gene amplified by PCR.

**
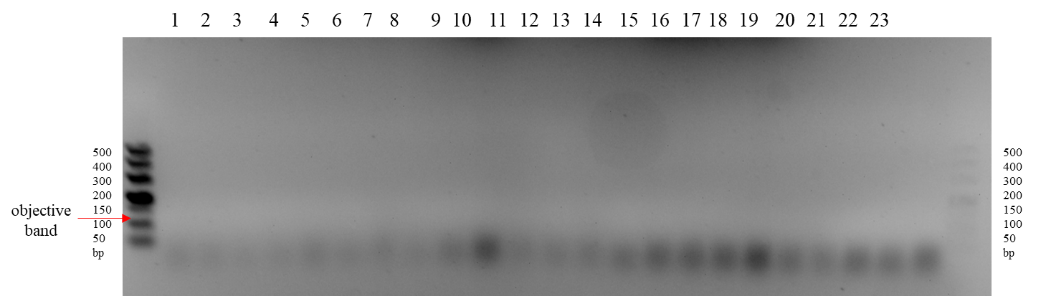
**

**
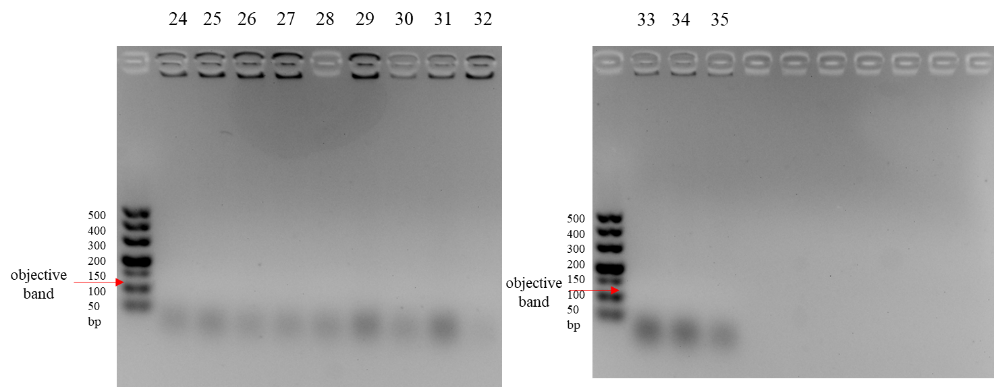
**

**Figure S6.** The results of agarose gel electrophoresis of MRSA isolated samples *pvl* target gene amplified by PCR.
